# Recurrence of primary extramedullary plasmacytoma in breast both simulating primary breast carcinoma

**DOI:** 10.1186/1477-7819-2-29

**Published:** 2004-08-31

**Authors:** Ahmad Kaviani, Mansoor Djamali-zavareie, Maryam Noparast, Sedigheh Keyhani-Rofagha

**Affiliations:** 1 Department of Surgery, Tehran University of Medical Sciences, Tehran, Iran; 2 Department of Pathology, Tehran University of Medical Sciences, Tehran, Iran; 3 Iranian Center for Breast Cancer, Tehran, Iran; 4 Professor of Pathology, Department of Pathology, Ohio State University, Ohio, USA; 5 Director, Iranian Center for Breast Cancer, Tehran, Iran

## Abstract

**Background:**

Extramedullary myelomas (plasmacytoma) are malignant proliferations of plasma cells in the absence of bone involvement. When they occur in the soft tissue they usually involve the upper respiratory tract and oral cavity. Extramedullary plasmacytomas of breast are uncommon.

**Case presentation:**

A 70 year-old woman with bilateral breast masses underwent excisional biopsy for suspected primary carcinoma that subsequently proved to be a recurrence from extramedullary plasmacytoma of the mediastinum. This was diagnosed and treated 5-years prior to appearance of breast lumps.

**Conclusion:**

Though uncommon, considering the possibility of metastatic carcinoma and primary, secondary or recurrent lymphoproliferative disease presenting as a breast mass may avoid unnecessary surgeries.

## Background

Extramedullary myelomas (plasmacytoma) are malignant proliferations of plasma cells in the absence of bone involvement. When occur in the soft tissue, it usually involve the upper respiratory tract and oral cavity [1]. Plasmacytomas of breast are very rare.

This report describes a patient with bilateral breast masses who underwent excision biopsy for suspected primary carcinoma that subsequently proved to be a recurrence from extramedullary plasmacytoma of mediastinum treated 5 years ago. To the best of our knowledge, this is the first case report of bilateral recurrence of a primary extramedullary plasmacytoma in breast tissues after a long disease-free interval.

## Case presentation

A 70 year-old woman with a one-month history of bilateral breast masses was referred to our cancer center for surgical evaluation. There was no associated breast pain, skin change or nipple discharge. There was no history of bone pain, weight loss, fatigue, fever or other systemic complaints and no family history of breast cancer. Significant past medical history included treatment for an extramedullary retrosternal plasmacytoma 5-years prior this admission.

At the time of the initial work-up for the retrosternal mass, immunoelectrophoresis showed no evidence for hyperproteinemia or paraproteinemia. Whole body bone scan was negative and a bone marrow biopsy revealed less than 5% of plasma cells. Therefore, multiple myeloma was excluded by nuclear medicine, laboratory and histology studies. The patient underwent radiation therapy (40 Gy with fraction size of 200 cGy delivered over 4 weeks) followed by chemotherapy with cyclophosphamide, cisplatin and prednisolone. The patient was followed by laboratory tests, chest roentgenography, and computed tomography annually. A bone scintigraphy was carried out after 2 years and showed no uptake patient was thereafter lost to follow-up.

Five years after initial diagnosis of extramedullary plasmacytoma the patient presented with bilateral breast masses. Physical examination revealed a 3.5 cm × 2.5 cm, mass in the upper inner quadrant of the right breast and a similar 5.0 cm × 4.5 cm mass in the lower inner quadrant of the left breast. No asymmetry, skin dimpling or signs of inflammation were present. There was no axillary or supraclavicular lymphadenopathy.

**Figure 1:**
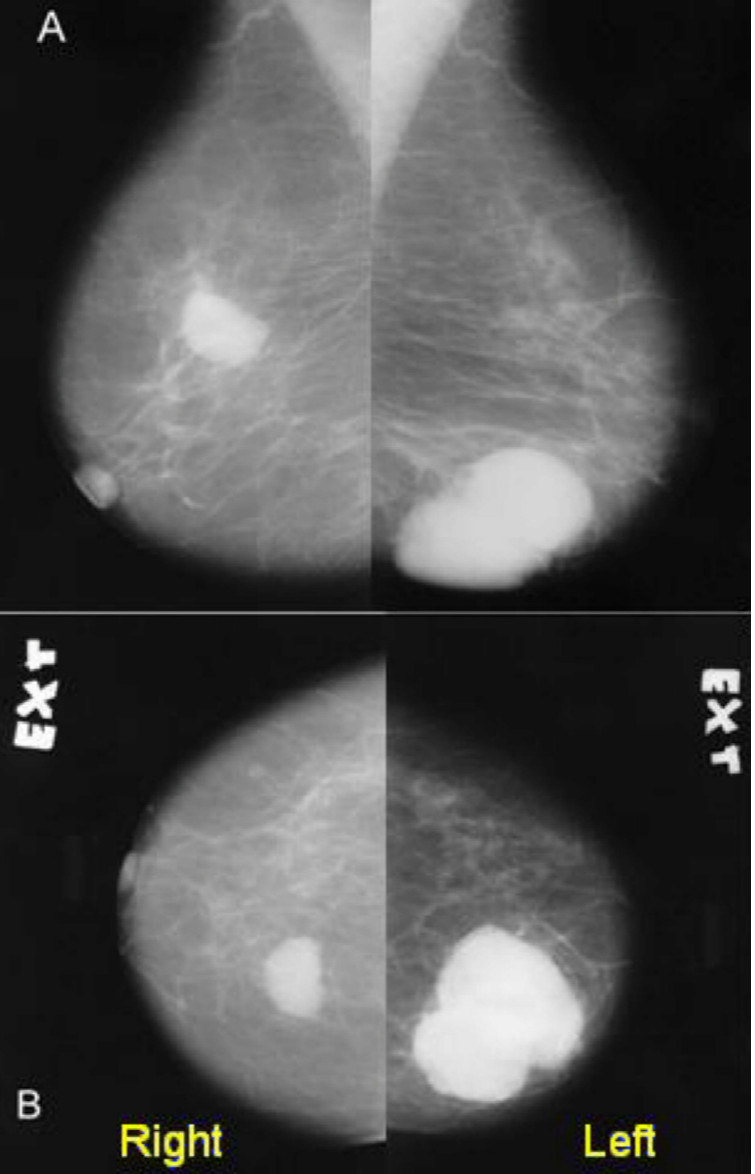
Mammography of the patients' breasts (A: mediolateral oblique, view B: craniocaudal view)

Mammography confirmed a well-defined 3.2 cm oval-shaped mass in the upper inner quadrant of the right breast, and a lobulated 5.5 cm density in lower inner quadrant of the left breast without any tissue distortion, inflammation and fibrotic reaction.(Figure 1) There were no microcalcification and satellite lesions. These masses were solid and hypoechoeic with multiple septations in sonography.

**Figure 2:**
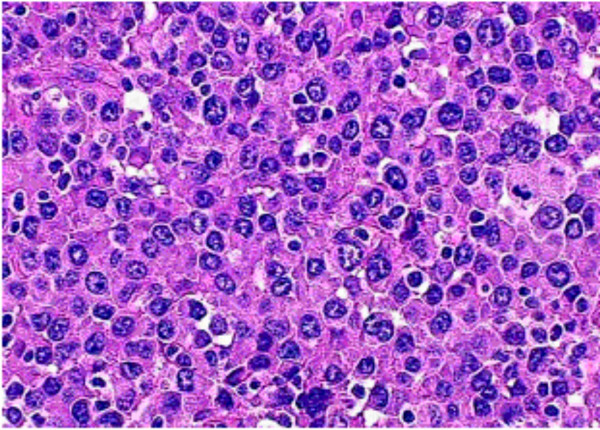
Photomicrograph showing nuclear pleomorphism, binucleated and multinucleated plasma cells with enlarged nucleoli (Hematoxylin & Eosin).

Excisional biopsy of the masses revealed a 5.0 (left) and 3.0 (right) well-defined, capsulated gritty mass surrounded by normal breast tissue. There was no extension from the capsulated masses to pectoral muscles or chest wall. Histopathological examination showed high-grade tumors composed of immature and mature plasma cells. Mitosis, necrosis, nuclear pleomorphism and binucleated and multinucleated plasma cells were seen. (Figure 2) Additional studies such as serum protein electrophoresis and immunoelectrophoresis were normal. No Bence Jones or other M components were detected in the urine. Skeletal surveys (Tc^99 ^bone scan and skull and pelvic X-rays) did not show any pathological changes. There was no evidence of anemia, hypercalcemia or renal insufficiency. However, the patient refused a second bone marrow biopsy.

**Figure 3:**
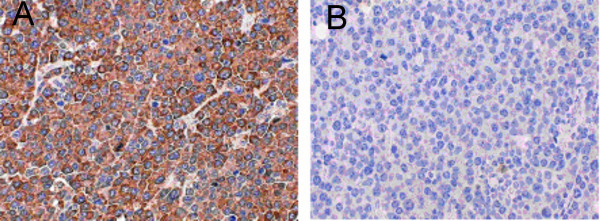
Lambda and kappa immunohistochemical stain showing strong and diffuse positivity for lambda (a) and negativity for kappa (b).

Immunohistochemical studies were performed on the paraffin embedded tissues to determine if the infiltrate had monoclonal character. The tumor cells were diffusely and strongly positive for lambda chains but negative for kappa chains. (Figure 3)

**Figure 4:**
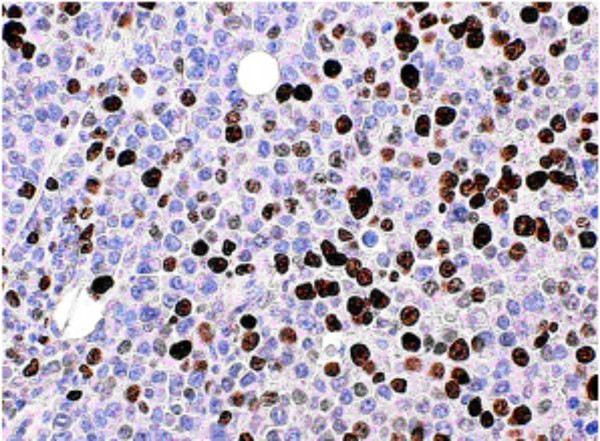
Ki67 immunohistochemical stain showing 50–80% nuclear positivity

The tumor cells were weakly positive for monoclonal mouse anti human placental V538C, and plasma cell markers (CD138). Nuclear prognostic marker (Ki67) showed 50% to 80% nuclear expression indicative of high proliferative activity and suggesting a plasmacytic tumor with anaplastic components (Figure 4). Other immunohistochemical stains including CD21, cytokeratin, S100, and HMB45 were negative.

A retrospective microscopic review of the mediastinal mass showed similar morphology to the breast tumor. Hence, the histological diagnosis of recurrent plasmacytoma was made.

The patient was treated with oral Melfalan and Prednisone. The patient has been disease free for twenty months after treatment and has showed no evidence of recurrence in the mediastinum, breast or any other region.

## Discussion

Primary soft tissue extramedullary plasmacytoma (SEP) is uncommon and is defined as a malignant tumor of plasma cells arising in the soft tissue in the absence of bone involvement. It can occur in any organ as a solitary form of plasma cell neoplasm [2]. Although SEP can arise throughout the body, almost 90% of the cases arise in the head and neck areas, most commonly in the upper respiratory tract including the nasal cavity, para nasal sinuses, oropharynx, salivary glands and larynx [3-7]. Several other sites can rarely be involved, including testis, bladder, urethra, breast, ovary, lung, pleura, thyroid, orbit, brain and skin tissues (1, 8–17). Approximately forty-five cases of breast plasmacytoma have been reported in published literature since 1928 (1, 2, 18–21). More than half of the lesions were unilateral (66%), with the majority of the cases occurring in the setting of multiple myeloma (77%) (1, 2, 18–20, 22).

When plasmacytoma originates from soft tissues, like the case presented here, the disease is usually associated with a relatively mild clinical behavior and long survival, suggesting that it is a truly different disease entity compared to other plasma cell tumors [23].

Dimopoulos *et al *(1999) reported that solitary extramedullary (soft tissue) plasmacytomas (SEP) are less common than solitary bone plasmocytoma (SBP), and have a better prognosis as the majority can be cured by local radiotherapy [24].

Liebross *et al *(1999) reported local recurrence rates of less than 5% after radiotherapy [6]. Mayr *et al *(1990) noted that the risk of distant relapse is more than 30%, which is significantly less than that seen with SBP [25]. Progressive disease may accrue as multiple myeloma, SBP or involvement of lymph nodes, skin or subcutaneous tissues. Its recurrence, if any, tends to be within 2–3 years of initial diagnosis. The reported ten year survival rate is at least 66% [3,7].

Involvement of the breast with SEP is uncommon and may occur either as a solitary primary tumor or as evidence of disseminated multiple myeloma [19]. Our patient was referred with bilateral breast masses. In an approach to a patient with bilateral breast masses, differential diagnosis includes: fibroadenomas, complex cysts, metastasis, lymphoma, synchronous breast cancer, focal fibrosis, fat necrosis, abscess, and phyllodes tumor [20]. Metastatic lesions of the breast from extramammary neoplasms are rare and in larger studies have been reported to constitute 0.4 to 2% of all breast malignancies. The most common are lymphomas and other tumors of hematological origin [26]. The striking feature in this case was the recurrence of an isolated plasmacytoma (which was treated successfully five year prior) in both breasts. As approximately 20% of patients who present with isolated extramedullary plasmacytoma will eventually develop multiple myeloma, close follow-up is strongly recommended [1,22].

Our patient did not have any prior breast aspiration cytology. There are many tumors, which may present with plasmacytoid appearance on aspiration cytology. Tumors such as small ductal carcinoma and lobular carcinoma of breast, metastatic carcinoid, metastatic melanoma and some lymphomas may represent with uncohesive group of cells with eccentric nuclei resembling plasma cells. Hence, a plasmacytoma (with anaplastic plasma cells) may be readily mistaken for carcinoma (or other undifferentiated neoplasm) not only clinically, but also on cytological examination. This would justify excision biopsy and the use of an extended immunohistochemical panel to include such markers as cytokeratin and S-100 in the assessments. To help the cytopathologist avoid misinterpretation, clinical history and presentation are extremely helpful. Most of the errors in histopathology and cytopathology diagnosis occur when pathologist is not aware of medical history of the patient and unusual clinical presentation.

This case emphasizes the importance of distinguishing a plasmacytoma of the breast from primary mammary carcinomas and other benign lesions to avoid unnecessary surgery and provide the appropriate treatment and adjuvant therapy.

## Authors' Contributions

AK carried out excision biopsies and drafted the manuscript.

MJZ did the histopathological examination and contributed to the pathological content of the manuscript.

SKR is the pathologist who confirmed the diagnosis and prepared the immunohistochemical stains and illustrations. He also contributed to pathological content of the manuscript

MN followed-up the patient and contributed to the manuscript preparation

All authors read and approved the final manuscript.
